# Supplementary material for: An enteric neuron-expressed variant ionotropic receptor detects ingested salts to regulate salt stress resistance
Source: bioRxiv. 2025 May 8:2025.04.11.648259. Preprint. [Version 3] doi: 10.1101/2025.04.11.648259 (PMC12087990; doi:10.1101/2025.04.11.648259)
Supplement: Supplement 1 [file NIHPP2025.04.11.648259v3-supplement-1.pdf]

bioRxiv preprint doi: https://doi.org/10.1101/2025.04.11.648259; this version posted May 8, 2025. The copyright holder for this preprint (which was not certified by peer review) is the author/funder, who has granted bioRxiv a license to display the preprint in perpetuity. It is made available under aCC-BY 4.0 International license.

Movie S1. (Right) 3D-rendered image of GLR-9::GFP localization at the I3 sensory ending (Left) generated using Imaris Analysis Software (version 10.2.0).

Movie S2. 3D reconstruction of the I3 sensory ending from electron microscope serial sections.

Data Table 1. List of upregulated and downregulated genes and gene categories across each condition/genotype from RNA-Seq analysis. The category list is ordered from highest to lowest gene count.

bioRxiv preprint doi: https://doi.org/10.1101/2025.04.11.648259; this version posted May 8, 2025. The copyright holder for this preprint (which was not certified by peer review) is the author/funder, who has granted bioRxiv a license to display the preprint in perpetuity. It is made available under aCC-BY 4.0 International license.

Table S1. List of strains used in this work.

Strain name	Genotype	Source/ Parent strain
PY12290	*glr-9::gfp*(*sy5669*); *oyEx761* [*glr-9*p::*myr-TagRfp*; *unc-122*p::*dsRed*]	Suny Biotech, This paper
PY12291	*oyEx762* [*glr-7*p::*glr-7*::*gfp*; *glr-9*p:: *myr-TagRfp; unc-122*p::*dsRed*]	This paper
PY12292	*glr-7(tm2877)*; *glr-9::gfp*(*sy5669*); *oyEx761* [*glr-9*p::*myr-TagRfp*; *unc-122*p::*dsRed*]	CGC, This paper
PY12293	*glr-9*(*oy180*); *oyEx762* [*glr-7*p::*glr-7*::*gfp*; *glr-9*p:: *myr-TagRfp; unc-122*p::*dsRed*]	This paper
PY12286	*oyIs98* [*glr-9*p::GCaMP6s; *unc-122*p::*dsRed*]	This paper
PY12294	*glr-9*(*oy180*); *oyIs98* [*glr-9*p::GCaMP6s; *unc-122*p::*dsRed*]	This paper
PY12295	*glr-9*(*oy180*); *oyIs98* [*glr-9*p::GCaMP6s; *unc-122*p::*dsRed*]; *oyEx763* [*glr-9*p::*glr-9*::*TagRfp*; *unc-122*p::*gfp*] Line-1	This paper
PY12296	*glr-9*(*oy180*); *oyIs98* [*glr-9*p::GCaMP6s; *unc-122*p::*dsRed*]; *oyEx764* [*glr-9*p::*glr-9*::*TagRfp*; *unc-122*p::*gfp*] Line-2	This paper
PY12297	*unc-119*(*ed3*); *otIs898* [*pha-4*prom2::3xNLS::GCaMP6s::*unc-54* 3' UTR]; *oyEx765* [*lgc-8*p::*mCherry*; *unc-122*p::*dsRed*]	OH18562, This paper
PY12298	*unc-119*(*ed3*); *otIs898* [*pha-4*prom2::3xNLS::GCaMP6s::*unc-54* 3' UTR]; *oyEx765* [*lgc-5*p::*mCherry*; *unc-122*p::*dsRed*]; *oyEx766* [*lgc-8*p::*glr-7*::*TagRfp*; *lgc-8*p::*glr-9*::*TagRfp*; *unc-122*p::*gfp*] Line-1	OH18562, This paper
PY12299	*unc-119*(*ed3*); *otIs898*[*pha-4*prom2::3xNLS::GCaMP6s::*unc-54* 3' UTR]; *oyEx765* [*lgc-5*p::*mCherry*; *unc-122*p::*dsRed*]; *oyEx767* [*lgc-8*p::*glr-7*::*TagRfp*; *lgc-8*p::*glr-9*::*TagRfp*; *unc-122*p:: *gfp*] Line-2	OH18562, This paper
PY12230	*glr-9*(*oy180*)	This paper
PY12228	*oyEx717* [*glr-9*p::*Chrimson::*T2A*::mCherry*; *unc-122*p::*gfp*] Line-1	This paper
PY12289	*glr-9*(*oy180*); *oyEx717* [*glr-9*p::*Chrimson::*T2A*::mCherry*; *unc-122*p::*gfp*]	This paper
PY12800	*loxP*::*unc-17*::*loxP* (*syb5779 syb5987*)	SWF1012, This paper
PY12801	*loxP*::*unc-17*::*loxP* (*syb5779 syb5987*); *oyEx768* [*glr-9*p::*Cre*::SL2::*gfp*; *unc-122*p::*gfp*]	SWF1012, This paper
PY12802	*egl-21*(*n476*); *kySi61* [*loxP::egl-21genomic::loxP*]	SWF717, This paper

bioRxiv preprint doi: https://doi.org/10.1101/2025.04.11.648259; this version posted May 8, 2025. The copyright holder for this preprint (which was not certified by peer review) is the author/funder, who has granted bioRxiv a license to display the preprint in perpetuity. It is made available under aCC-BY 4.0 International license.

PY12803	*egl-21*(*n476*); *kySi61* [*loxP::egl-21genomic::loxP*]; *oyEx768* [*glr*-9p::*Cre*::SL2::*gfp*; *unc-122*p::*gfp*]	SWF717, This paper
VC2324	*flp-6*(*ok3056*)	CGC
PY12804	*glr-9(oy180)*; *flp-6*(*ok3056)*	This paper
PY12288	*loxP::flp-6::loxP(oy203)*	This paper
PY12805	*loxP::flp-6*::*loxP*(*oy203); oyEx768* [*glr-*9p::*Cre*::SL2::*gfp*; *unc-122*p::*gfp*]	This paper
RJP5269	*unc-31* (*rp166*[GFP::TEV::AID::FLAG::*unc-31*])	CGC
PY12806	*unc-31* (*rp166*[GFP::TEV::AID::FLAG::*unc-31*]); *oyEx769* [*glr-9*p::TIR1::*mScarlet*; *unc-122*p::*gfp*] Line-1	This paper
PY12807	*unc-31* (*rp166*[GFP::TEV::AID::FLAG::*unc-31* *oyEx769* [*glr-9*p::TIR1::SL2::*mScarlet*; *unc-122*p::*gfp*] Line-2	This paper
PY12808	*unc-13(e51); oyIs98* [*glr-9*p::GCaMP6s; *unc-122*p::*dsRed*]	This paper
PY12809	*unc-31(e169); oyIs98* [*glr-9*p::GCaMP6s; *unc-122*p::*dsRed*]	This paper
PY12810	*tax-4*(*p678*); *oyIs98* [*glr-9*p::GCaMP6s; *unc-122*p::*dsRed*]	This paper
PY12811	*osm-9*(*oy202*); *oyIs98* [*glr-9*p::GCaMP6s; *unc-122*p::*dsRed*]	This paper
PY12812	*che-1*(*p674*); *oyIs98* [*glr-9*p::GCaMP6s; *unc-122*p::*dsRed*]	This paper
PY12813	*oyIs97* [*flp-6*p::GCaMP; *unc-122*p::*gfp*]; *oyEx770* [*che-1*p::*glr-9*::*TagRfp; che-1*p::*glr-7*::*TagRfp*; *unc-122*p::*dsRed*]	This paper
PR678	*tax-4*(*p678*)	CGC
CX10	*osm-9*(*ky10)*	CGC
CX4544	*ocr-2*(*ak47*)	CGC
FX30280	*flp-5*(*tm10075*)	NBRP
FX02427	*flp-13*(*tm2427*)	NBRP
AX1410	*flp-18*(*db99*)	CGC
FX02179	*flp-18*(*tm2179*)	NBRP
RB2269	*flp-34*(*ok3071*)	CGC
FX03023	*nlp-3*(*tm3023*)	NBRP
TM2569	*nlp-7*(*tm2984*)	NBRP
FX06232	*nlp-10*(*tm6232*)	NBRP
PHX530	*nlp-11*(*syb530*)	CGC
FX02569	*nlp-21*(*tm2569*)	NBRP
PS8789	*dmsr-1*(*sy1522*)	CGC
PS8854	*dmsr-7*(*sy1539*)	CGC
PS8483	*frpr-8*(*sy1362*)	CGC
MT14666	*egl-6*(*n4537*)	CGC

bioRxiv preprint doi: https://doi.org/10.1101/2025.04.11.648259; this version posted May 8, 2025. The copyright holder for this preprint (which was not certified by peer review) is the author/funder, who has granted bioRxiv a license to display the preprint in perpetuity. It is made available under aCC-BY 4.0 International license.

Table S2. List of plasmids used in this work.

**Plasmid name**	**Description**	**Source**
PSAB1374	*glr-9*p::*myr-TagRfp*	This paper
PSAB1373	*glr-7*p::*glr-7*::*gfp*	This paper
PSAB1383	*glr-9*p::GCaMP6s	This paper
PSAB1375	*glr-9*p::*glr-9*::*TagRfp*	This paper
PSAB1376	*lgc-8*p::*mCherry*	This paper
PSAB1378	*lgc-*8p::*glr-7*::*TagRfp*	This paper
PSAB1377	*lgc-*8p::*glr-*9::*TagRfp*	This paper
PSAB1371	*glr-9p::Chrimson::T2A::mCherry*	This paper
PSAB1379	*glr-*9p::*Cre*::*SL2*::*gfp*	This paper
PSAB1380	*glr-9*p::TIR1::*mScarlet*	This paper
PSAB1381	*che-1*p::*glr-7*::*TagRfp*	This paper
PSAB1382	*che-1*p::*glr-9*::*TagRfp*	This paper

bioRxiv preprint doi: https://doi.org/10.1101/2025.04.11.648259; this version posted May 8, 2025. The copyright holder for this preprint (which was not certified by peer review) is the author/funder, who has granted bioRxiv a license to display the preprint in perpetuity. It is made available under aCC-BY 4.0 International license.

REFERENCES

Albrecht, D.R., and Bargmann, C.I. (2011). High-content behavioral analysis of *Caenorhabditis elegans* in precise spatiotemporal chemical environments. Nat Methods *8*, 599-605.

Benton, R., Vannice, K.S., Gomez-Diaz, C., and Vosshall, L.B. (2009). Variant ionotropic glutamate receptors as chemosensory receptors in *Drosophila*. Cell *136*, 149-162.

Dodd, W., Tang, L., Lone, J.C., Wimberly, K., Wu, C.W., Consalvo, C., Wright, J.E., Pujol, N., and Choe, K.P. (2018). A damage sensor associated with the cuticle coordinates three core environmental stress responses in *Caenorhabditis elegans*. Genetics *208*, 1467-1482.

Khan, M., Hartmann, A.H., O'Donnell, M.P., Piccione, M., Pandey, A., Chao, P.-H., Dwyer, N.D., Bargmann, C.I., and Sengupta, P. (2022). Context-dependent reversal of odorant preference is driven by inversion of the response in a single sensory neuron type. PLoS Biol *20*, e3001677.

Rohlfing, A.K., Miteva, Y., Moronetti, L., He, L., and Lamitina, T. (2011). The *Caenorhabditis elegans* mucin-like protein OSM-8 negatively regulates osmosensitive physiology via the transmembrane protein PTR-23. PLoS Genet *7*, e1001267.

Taylor, S.R., Santpere, G., Weinreb, A., Barrett, A., Reilly, M.B., Xu, C., Varol, E., Oikonomou, P., Glenwinkel, L., McWhirter, R., *et al.* (2021). Molecular topography of an entire nervous system. Cell *184*, 4329-4347 e4323.

Zheng, Y., Brockie, P.J., Mellem, J.E., Madsen, D.M., and Maricq, A.V. (1999). Neuronal control of locomotion in *C. elegans* is modified by a dominant mutation in the GLR-1 ionotropic glutamate receptor. Neuron *24*, 347-361.

Zuo, J., De Jager, P.L., Takahashi, K.A., Jiang, W., Linden, D.J., and Heintz, N. (1997). Neurodegeneration in Lurcher mice caused by mutation in delta2 glutamate receptor gene. Nature *388*, 769-773.

bioRxiv preprint doi: https://doi.org/10.1101/2025.04.11.648259; this version posted May 8, 2025. The copyright holder for this preprint (which was not certified by peer review) is the author/funder, who has granted bioRxiv a license to display the preprint in perpetuity. It is made available under aCC-BY 4.0 International license.

a

IR-like protein	TM #	Extended amino-terminal domain	Ala in TMIII	Arg in S1 domain	Thr in S2 domain	Asp or Glu in S2 domain
GLR-7	3	Yes	Yes	Yes	No	Yes
GLR-8	3	No	Yes	Yes	No	No
GLR-9	3	No	No	Yes	No	Yes
GLR-10	3	No	No	Yes	No	No
ZK867.2	3	No	Yes	Yes	No	No
F59E12.8	3	No	No	Yes	No	No
T25E4.2	3	No	No	Yes	No	No

b

IR-like gene	Expression In:	
	Pharyngeal Enteric Neurons	Somatic Neurons
glr-7	I3, I6, M3, NSM	PLM, ALM, PVM
glr-8	I6, NSM, I1, MI, MC, M3, I3, M5, I2	PVD, PHB, PHA, IL2, AVL, URB, PLM, ALM, ADL, AVM
glr-9	I3	none
glr-10	I6, NSM	none
ZK867.2	I6, NSM	PQR, PHA, PVD, AQR, ADL, AUA, AVH, URY, PHC, ASK, PHB, OLQ, URX, AVF, PVT, RIH
F59E12.8	none	none
T25E4.2	NSM, I6, MI	none

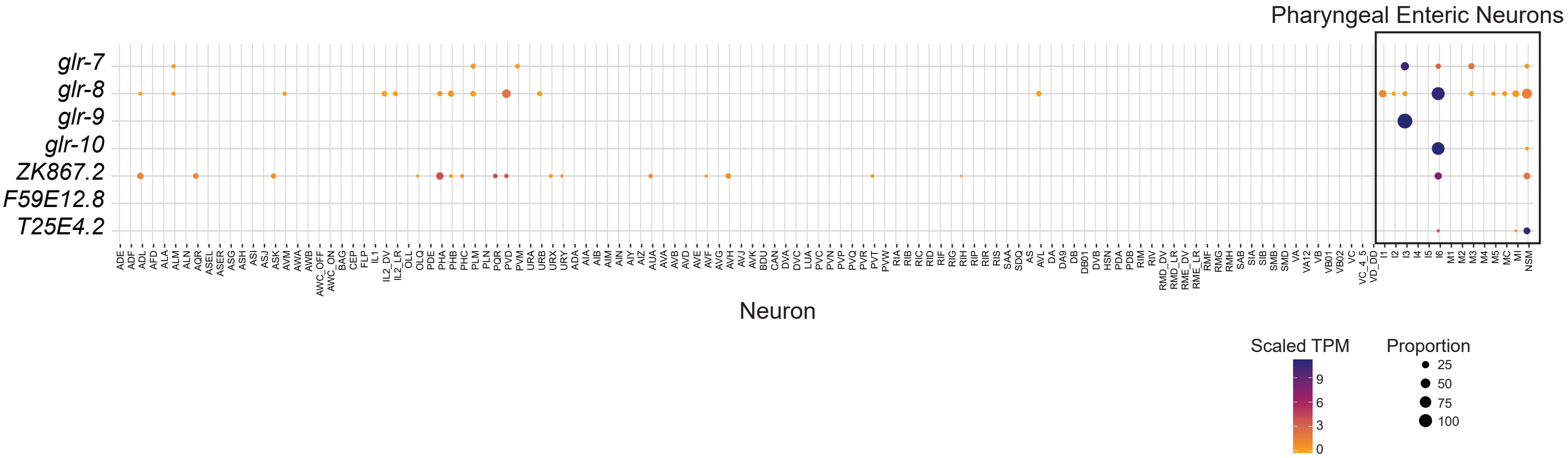

Yeon et al
Figure S1

bioRxiv preprint doi: https://doi.org/10.1101/2025.04.11.648259; this version posted May 8, 2025. The copyright holder for this preprint (which was not certified by peer review) is the author/funder, who has granted bioRxiv a license to display the preprint in perpetuity. It is made available under aCC-BY 4.0 International license.

Fig. S1. *C. elegans* IR-like genes are expressed highly or exclusively in PENs.

a) Features of IR-like proteins in *C. elegans*. TM: transmembrane domain. Mutating the conserved Ala in TMIII in ionotropic glutamate receptors results in a constitutively open channel (Zheng et al., 1999; Zuo et al., 1997). The Arg, and Thr and Asp/Glu residues, in the S1 and S2 ligand-binding domains, respectively, directly interact with glutamate or synthesized agonists in ionotropic glutamate receptors (Benton et al., 2009).

b) Predicted expression of IR-like genes adapted from the *C. elegans* neuronal gene expression map and network (CeNGEN) (Taylor et al., 2021).

bioRxiv preprint doi: https://doi.org/10.1101/2025.04.11.648259; this version posted May 8, 2025. The copyright holder for this preprint (which was not certified by peer review) is the author/funder, who has granted bioRxiv a license to display the preprint in perpetuity. It is made available under aCC-BY 4.0 International license.

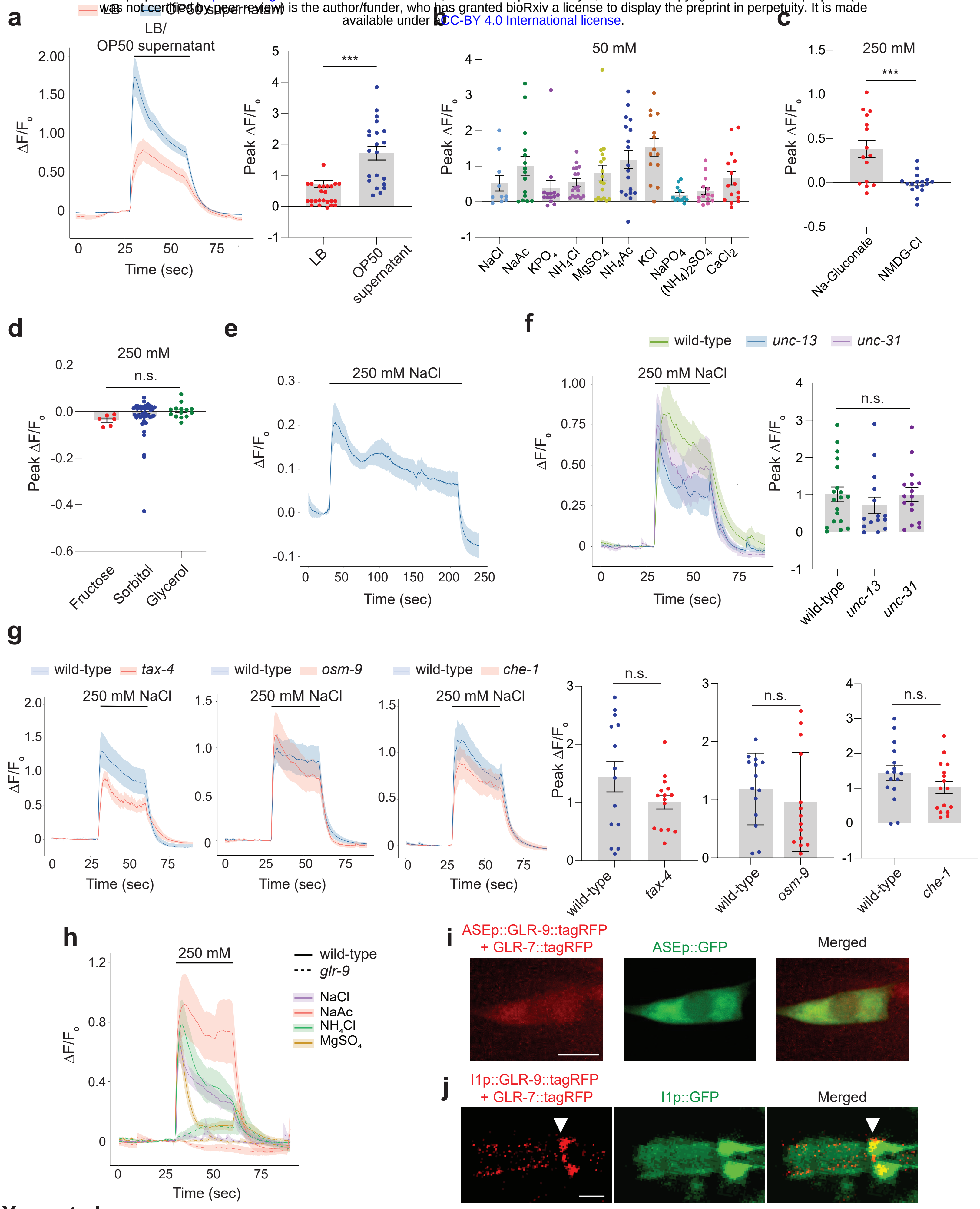

Yeon et al
Figure S2

bioRxiv preprint doi: https://doi.org/10.1101/2025.04.11.648259; this version posted May 8, 2025. The copyright holder for this preprint (which was not certified by peer review) is the author/funder, who has granted bioRxiv a license to display the preprint in perpetuity. It is made available under aCC-BY 4.0 International license.

Fig. S2. I3 responds cell-autonomously to multiple cations via GLR-9.

a) Average (left) and peak intensity changes (right) of GCaMP6s fluorescence in wild-type I3 neurons in response to a 30 sec pulse of LB alone or supernatant of an OP50 bacterial culture grown in LB. *** indicates different at P<0.001 (t-test).

b-d) Peak fluorescence intensity changes in I3 in response to the indicated chemical concentrations. Average traces are shown in Fig. 2a-c. *** indicates different at P<0.001 (c: t-test; d: one-way ANOVA and Dunnett's test).

e) Mean GCaMP6s fluorescence change in I3 in response to a 3 min pulse of 250 mM NaCl.

f,g) Average (left) and peak intensity changes (right) of GCaMP6s fluorescence in I3 in response to a 30 sec pulse of 250 mM NaCl in animals of the indicated genotypes. Alleles used were *unc-13(e51), unc-31(e169)*, *tax-4(p678), osm-9(oy202),* and *che-1(p674).*

h) Mean GCaMP6s fluorescence changes in response to a 30 sec pulse of the indicated salts at 250 mM in wild-type and *glr-9(oy180)* mutants. n>17 each. Wild-type data were interleaved with data in Fig. 2d-g, and are repeated.

i,j) Representative images of GLR-9/GLR-7::tagRFP localization in ASE soma (i), and I1 (j). Expression in ASE and I1 was driven under the *che-1* and *lgc-8* promoters, respectively. Arrowhead indicates the non-ciliated sensory ending of I1 in j. Anterior is at left in all images. Scale bar: 5 μm.

Shaded regions in all traces indicate SEM. Each dot in the scatter plots is the value from a single neuron. Horizontal lines in scatter plots indicate the mean; errors are SEM. Data shown are from 2-3 independent experiments each. n.s.- not significant.

bioRxiv preprint doi: https://doi.org/10.1101/2025.04.11.648259; this version posted May 8, 2025. The copyright holder for this preprint (which was not certified by peer review) is the author/funder, who has granted bioRxiv a license to display the preprint in perpetuity. It is made available under aCC-BY 4.0 International license.

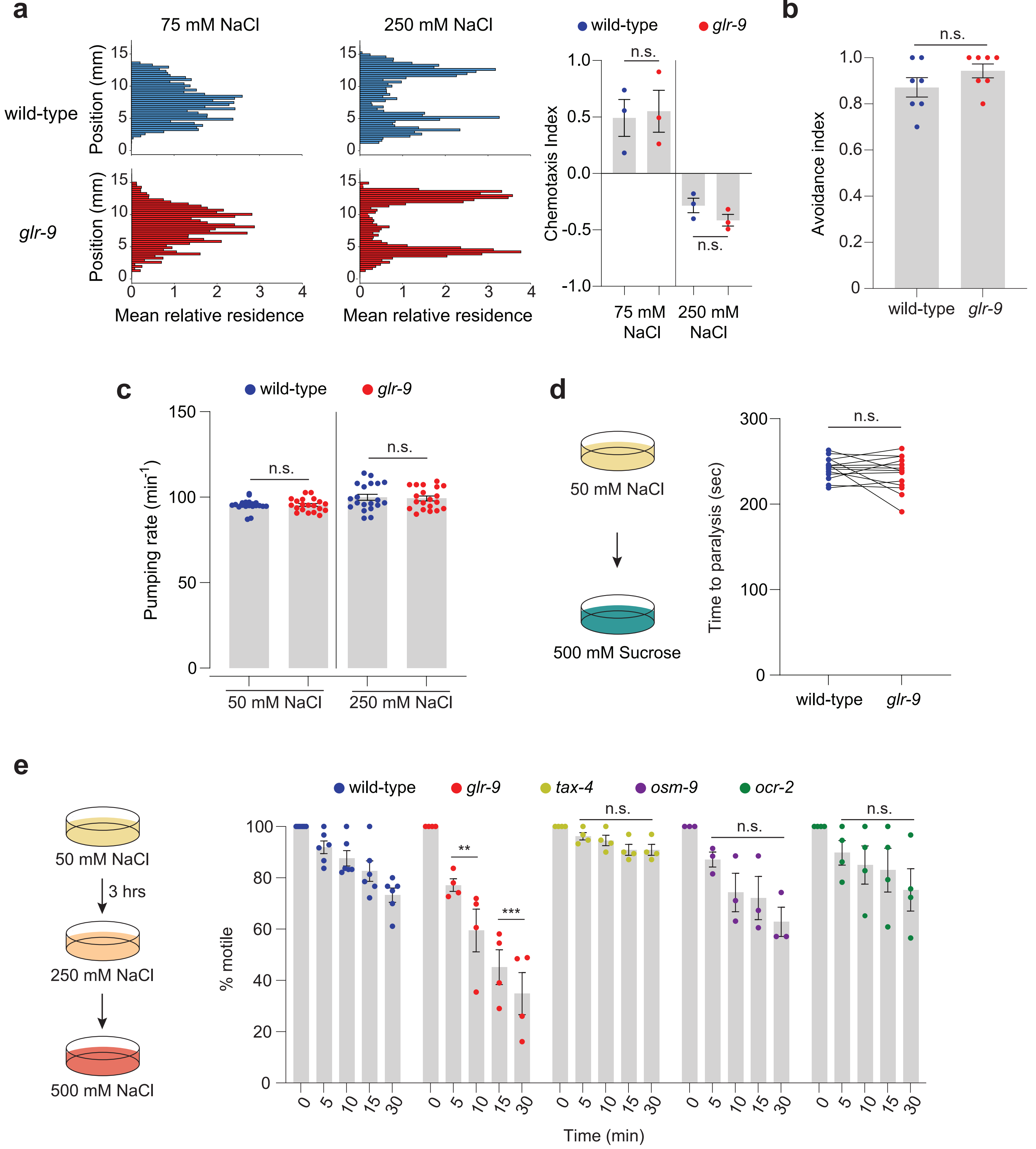

Yeon et al
Figure S3

bioRxiv preprint doi: https://doi.org/10.1101/2025.04.11.648259; this version posted May 8, 2025. The copyright holder for this preprint (which was not certified by peer review) is the author/funder, who has granted bioRxiv a license to display the preprint in perpetuity. It is made available under aCC-BY 4.0 International license.

Fig. S3. *glr-9* mutants do not exhibit altered behavioral responses to salt.

a) (Left) Histograms showing average relative residence of wild-type and *glr-9(oy180)* animals in a microfluidics behavioral arena (Albrecht and Bargmann, 2011; Khan et al., 2022) with a central stripe of the indicated salt concentrations. (Right) Chemotaxis indices quantified from the microfluidics behavioral assays. Each dot is the index from a single assay of ~15 animals each.

b) Percentage of animals of the indicated genotypes remaining within the glycerol ring after 10 mins. Each dot is the value from a single assay of 10 animals each.

c) Pumping rate on bacteria-seeded plates containing the indicated salt concentrations. Each dot is the measurement from a single animal.

d) Quantification of time to paralysis of wild-type or *glr-9* mutants in the shown assay conditions (cartoons at left). Each dot is the time at which all ten animals in a single assay are immotile. Wild-type and *glr-9* mutants were examined in parallel in the same assay.

e) Percentage of animals of the indicated genotypes that are motile at each time point following a shift from the acclimation plate to the assay plate. Alleles used were *glr-9(oy180), tax-4(p678), osm-9(ky10)*, and *ocr-2(ak47)*. Each dot is the value from a single assay; n=30 animals per assay. ** and *** indicate different from wild-type at the corresponding time at P<0.01 and 0.001, respectively (one-way ANOVA and Dunnett's test).

Horizontal lines in all scatter plots indicate the mean; errors are SEM. n.s. – not significant.

bioRxiv preprint doi: https://doi.org/10.1101/2025.04.11.648259; this version posted May 8, 2025. The copyright holder for this preprint (which was not certified by peer review) is the author/funder, who has granted bioRxiv a license to display the preprint in perpetuity. It is made available under aCC-BY 4.0 International license.

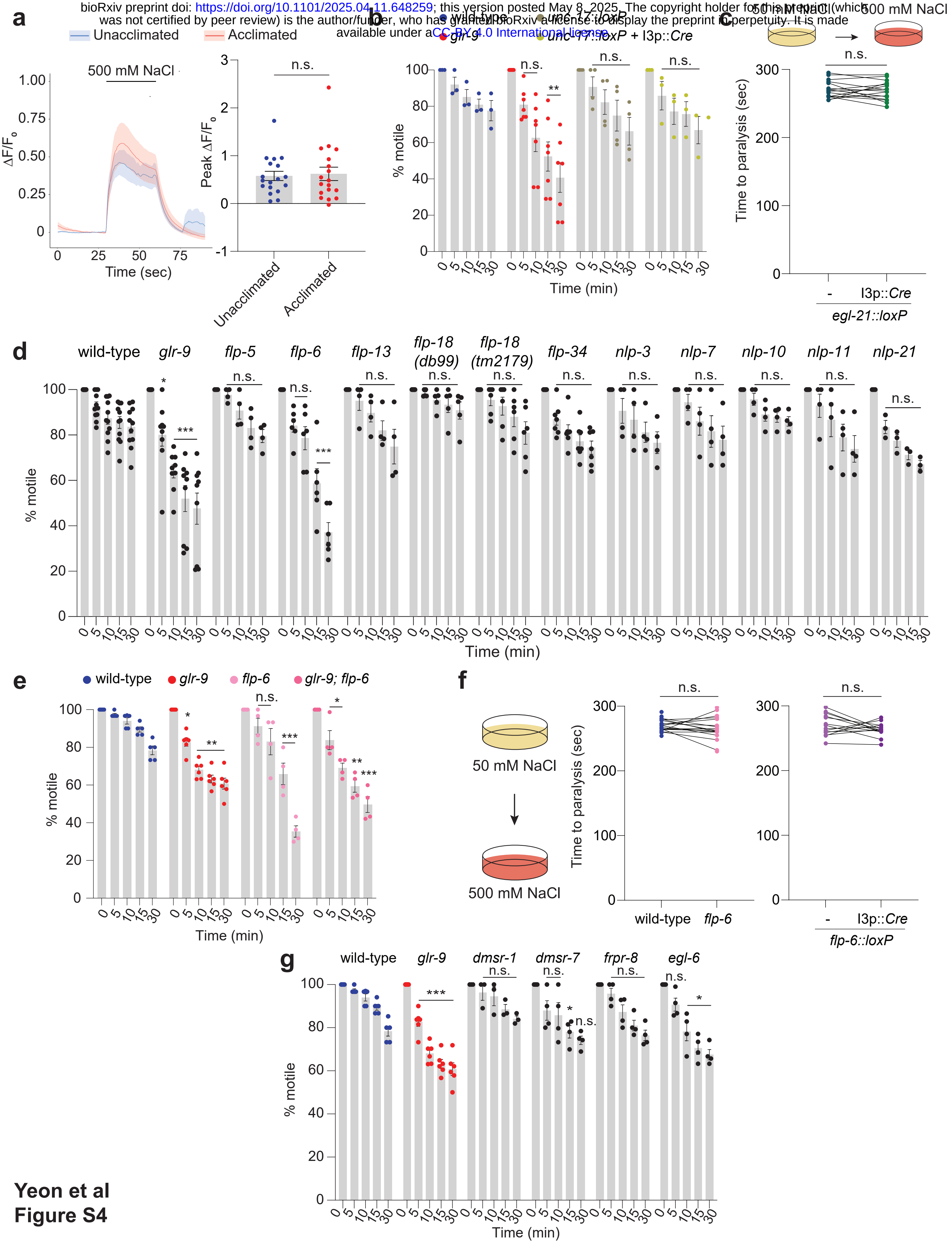

Yeon et al
Figure S4

bioRxiv preprint doi: https://doi.org/10.1101/2025.04.11.648259; this version posted May 8, 2025. The copyright holder for this preprint (which was not certified by peer review) is the author/funder, who has granted bioRxiv a license to display the preprint in perpetuity. It is made available under aCC-BY 4.0 International license.

Fig. S4. Peptidergic but not cholinergic signaling from I3 is required for salt acclimation.

a) (Left) Mean GCaMP6s fluorescence change in I3 in response to 500 mM NaCl prior to and following acclimation to 250 mM NaCl for 3 hrs. (Right) Peak fluorescence intensity changes calculated from traces on the left. Each dot is the value from a single I3 neuron.

b,d,e,g) Percentage of animals of the indicated genotypes that are motile at each time point following a shift from the acclimation plate (250 mM NaCl for 3 hrs) to 500 mM NaCl. Alleles used were *flp-5(tm10075), flp-6(ok3056), flp-13(tm2427)*, *flp-18(db99), flp-18(tm2179), flp-34(ok3071), nlp-3(tm3023), nlp-7(tm2984), nlp-10(tm6232), nlp-11(syb530), nlp-21(tm2569), dmsr-1(sy1522), dmsr-7(sy1539), frpr-8(sy1362)* and *egl-6(n4537)*. Cre was expressed in I3 under the *glr-9* promoter. Each dot is the value from a single assay; n=30 animals per assay. *, ** and *** indicate different from wild-type at the corresponding time at $P<0.05$, 0.01, and 0.001, respectively (one-way ANOVA and Dunnett's test). For e and g, wild-type and *glr-9* data were interleaved, and are repeated.

c,f) Quantification of time to paralysis of animals of the indicated genotypes in the shown assay conditions (cartoons at top (c) or left (f)). Each dot is the time at which all ten animals in a single assay are immotile. Cre was expressed in I3 under the *glr-9* promoter. Control and experimental animals were examined in parallel in the same assay.

The *glr-9(oy180)* allele was used in all experiments. Horizontal lines in all scatter plots indicate the mean; errors are SEM. Data shown are from 2-3 independent experiments each. n.s. - not significant.

bioRxiv preprint doi: https://doi.org/10.1101/2025.04.11.648259; this version posted May 8, 2025. The copyright holder for this preprint (which was not certified by peer review) is the author/funder, who has granted bioRxiv a license to display the preprint in perpetuity. It is made available under aCC-BY 4.0 International license.

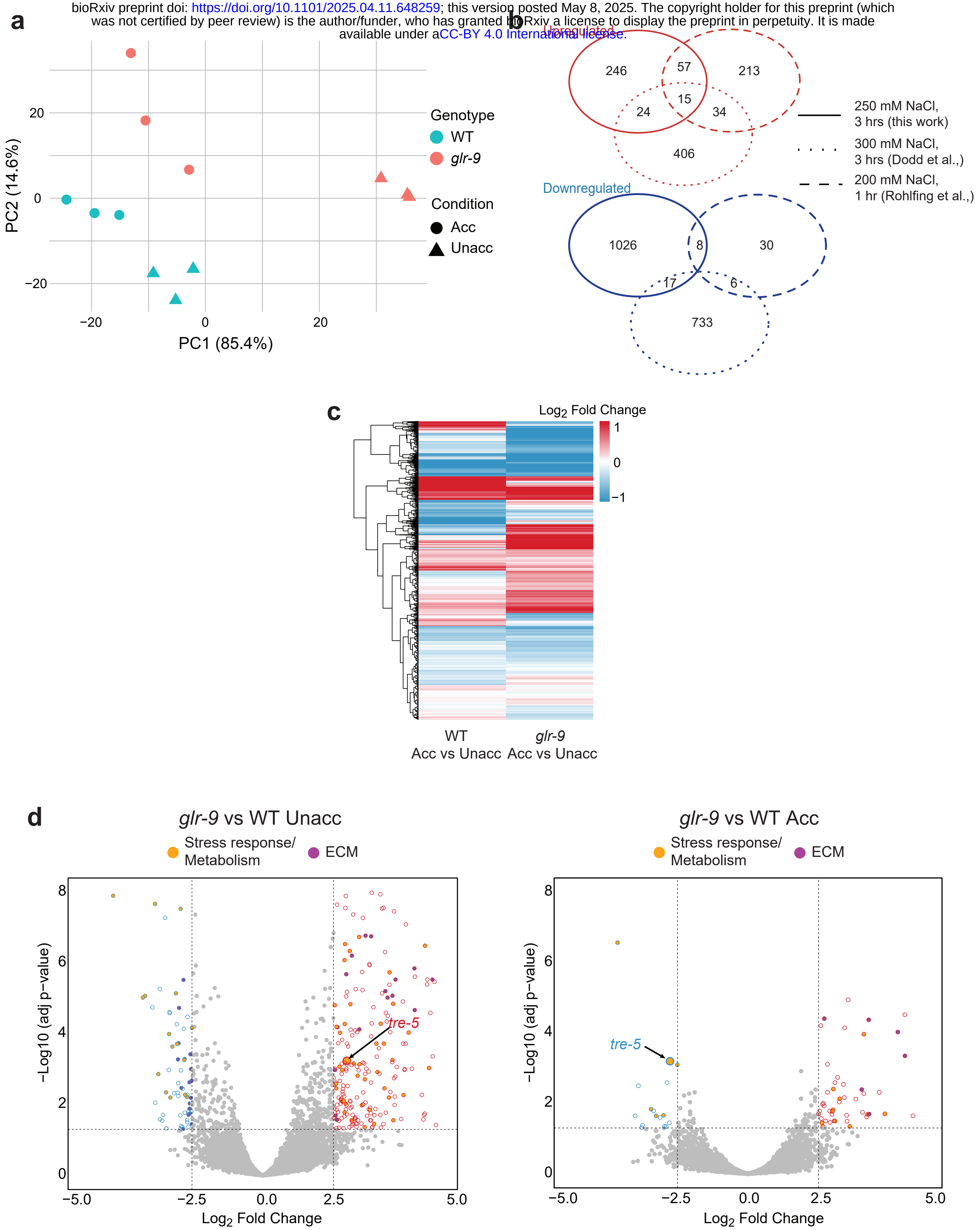

Yeon et al
Figure S5

bioRxiv preprint doi: https://doi.org/10.1101/2025.04.11.648259; this version posted May 8, 2025. The copyright holder for this preprint (which was not certified by peer review) is the author/funder, who has granted bioRxiv a license to display the preprint in perpetuity. It is made available under aCC-BY 4.0 International license.

Fig. S5. Salt acclimation-induced changes in gene expression are partly regulated by GLR-9-mediated signaling.

a) Principal component analysis plot of RNA-Seq data clustered by genotype and condition. Each data point is a single replicate.

b) Venn diagram showing overlap of differentially expressed genes between unacclimated and salt acclimated wild-type animals identified in this and previously published work (Dodd et al., 2018; Rohlfing et al., 2011).

c) A hierarchically clustered heatmap of all differentially expressed genes between acclimated and unacclimated wild-type and *glr-9* mutants.

d) Volcano plots of differential gene expression between unacclimated and salt-acclimated wild-type and *glr-9(oy180)* animals. Horizontal and vertical dashed lines indicate significant at adjusted p-value <0.05 and $\log_2$ fold change >2 or <−2, respectively. A subset of genes involved in osmotolerance and discussed in this work is indicated. Molecules categorized as being involved in stress responses or metabolism, and in extracellular matrix remodeling, are indicated in yellow and purple, respectively.

bioRxiv preprint doi: https://doi.org/10.1101/2025.04.11.648259; this version posted May 8, 2025. The copyright holder for this preprint (which was not certified by peer review) is the author/funder, who has granted bioRxiv a license to display the preprint in perpetuity. It is made available under aCC-BY 4.0 International license.

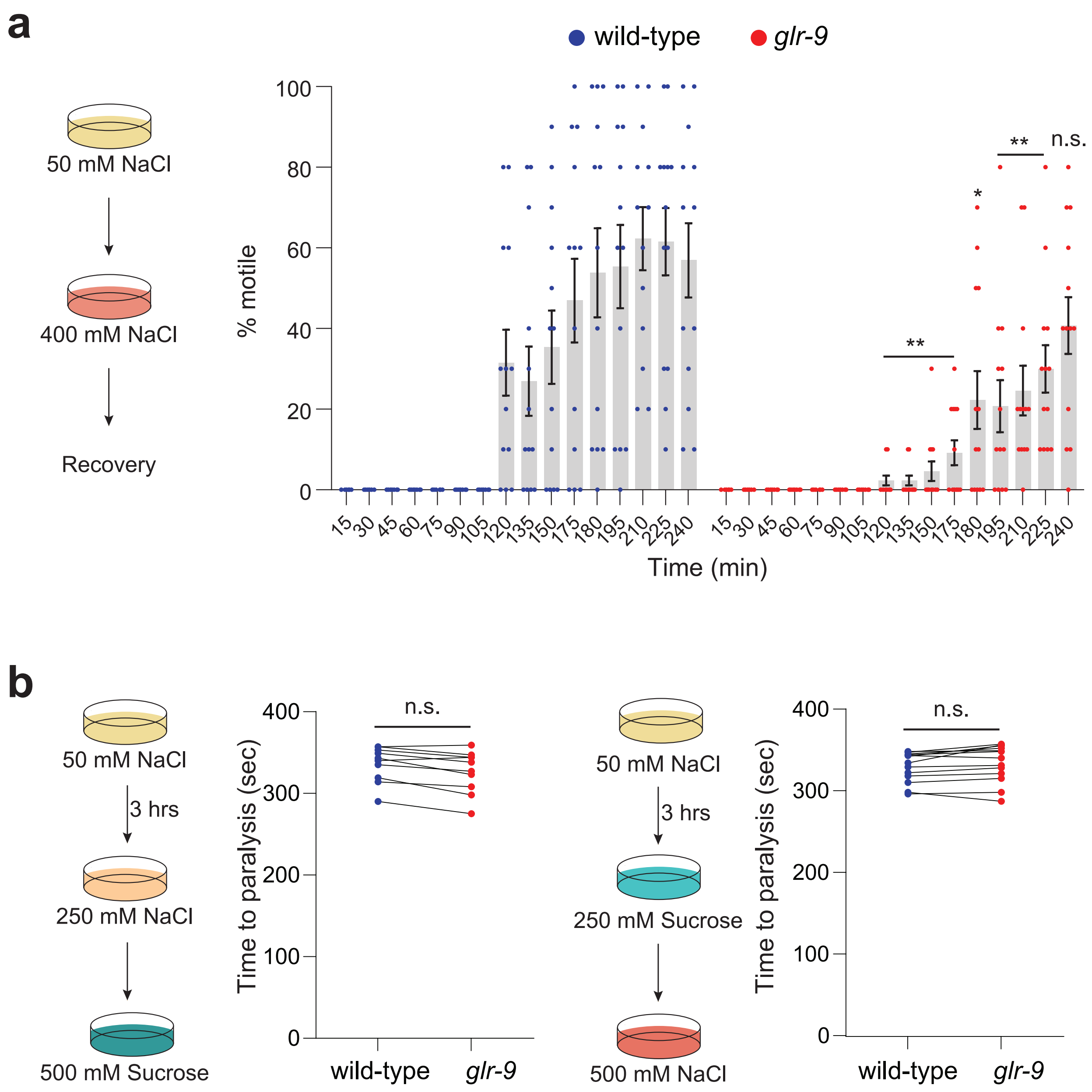

Yeon et al
Figure S6

bioRxiv preprint doi: https://doi.org/10.1101/2025.04.11.648259; this version posted May 8, 2025. The copyright holder for this preprint (which was not certified by peer review) is the author/funder, who has granted bioRxiv a license to display the preprint in perpetuity. It is made available under aCC-BY 4.0 International license.

Fig. S6. *glr-9* mutants exhibit reduced recovery following salt stress.

a) Percentage of wild-type or *glr-9(oy180)* mutants that are motile at each time point after being moved from 50 mM NaCl to 400 mM NaCl. Each dot is the value from a single assay; n=10 animals per assay. * and ** indicate different from wild-type at the corresponding time at P<0.05, 0.01, and 0.001, respectively (one-way ANOVA and Dunnett's test). n.s. – not significant.

b) Quantification of time to paralysis upon shifting wild-type or *glr-9(oy180)* mutants acclimated to 250 mM NaCl or 250 mM sucrose to 500 mM sucrose or 500 mM NaCl, respectively. Each dot is the time at which all ten animals in a single assay are immotile. Wild-type and *glr-9* mutants were examined in parallel in the same assay. n.s. – not significant.
